# Design and implementation of RESCUR in Sweden for promoting resilience in children: a study protocol

**DOI:** 10.1186/s12889-018-6145-7

**Published:** 2018-11-12

**Authors:** Charli Eriksson, Birgitta Kimber, Therése Skoog

**Affiliations:** 1 Department of Public Health Science, Stockholm University, Stockholm, Sweden; 2 Department of Clinical Sciences, Umeå University, Umeå, Sweden; 3 Department of Psychology, Göteborg University, Gothenburg, Sweden

**Keywords:** Promotion, Resilience, Children, Controlled trial, Intervention, Implementation

## Abstract

**Background:**

This research program aims to investigate the implementation and effects of a theoretically promising prevention method. It is being developed in a European research collaboration within a Comenius project (2012–2015) between 6 European universities (in Malta, Italy, Greece, Croatia, Portugal and Sweden) with the purpose of enhancing European children’s resilience.

**Methods/design:**

RESCUR in Sweden consists in a RCT study of the *Resilience Curriculum* (*RESCUR*) that is taking place in Sweden 2017–2019. The study is being performed by Junis, IOGT-NTO’s Junior Association, part of IOGT International, in conjunction with researchers at Göteborg, Umeå and Stockholm universities, and is being funded by the Public Health Agency of Sweden.

Around 1000 children of the ages 7–12 will, through their schools and associations, or via groups in social services, be acquainted with the material. Children will learn and practice mindfulness, storytelling, group discussions and much more, all designed to strengthen protective factors and increase their resilience. The program also involves parents, who are taking part in the work to reinforce children’s protective factors.

Based on the work with groups of children, an effectiveness study including children aged 7–12 in school classes, with randomized and controlled pre- and post-measurements, self-rating questionnaires and group observations is being performed. The program will also be implemented in a non-governmental organization and in groups in social services. The study also investigates forms of implementation.

**Discussion:**

The design of the study will enable the researchers to answer five research questions by using a mixed-methods approach. Implementation will be studied, which is a necessary prerequisite for an effect study. Moreover, the research procedure has been tailored to the target group, with age-appropriate measures as well as multiple informants, which will produce high-quality data for analysis. A special ethical challenge is the study of young children, and efforts to give children a voice have been included in the program. This project is regarded as having good potential to benefit children in general, and particularly children in vulnerable positions.

**Trial registration:**

National Institute of Health, ClinicalTrials.gov identifier NCT03655418. Registered August 31, 2018.

## Background

A resilience curriculum to foster the psychosocial development of children in early and primary education has been developed as a direct response to the current social and economic situation in Europe. The curriculum seeks specifically to promote the academic, emotional and social learning of children who may be at risk of early school-leaving, absenteeism, school failure, social exclusion and mental health problems, by providing them with key tools to overcome disadvantages and obstacles in their development whilst making use of their strengths [1, 2].

### Top of form

This research program aims to investigate the implementation and effects of a theoretical promising method developed in a European research collaboration within a Comenius project (2012–2015) between 6 European universities (in Malta, Italy, Greece, Croatia, Portugal and Sweden). The program has the purpose of enhancing European children’s resilience [1, 2]. RESCUR in Sweden consists in a RCT study of the *Resilience Curriculum* (*RESCUR*) that is taking place in Sweden 2017–2019. The study is being performed by a non-governmental organization (Junis, IOGT-NTO’s Junior Association, part of International Organization of Good Templars International) and researchers at Göteborg, Umeå and Stockholm universities. The focus is on children in general as well as children exposed to different social risks. They may be refugees or relatives of a person with harmful use of alcohol. In the Swedish study, we are also studying the method among children in general. It is intended that the study’s findings are will be compared with those of other impact studies planned in the other European RESCUR partner countries, such as Portugal and Italy.

### Resilience

The concept of resilience is central, and refers to the ability to cope with crises, changes and stressors without breaking down [1]. Most children who are exposed to stressful environments will develop positively despite the odds against them. These children, e.g., children of schizophrenic parents who did not get ill, were previously described as invulnerable, [3, 4]. Research has shown that a key factor is resilience, where the interaction between risk and protective factors results in different development patterns [5–7]. The approach means a shift from a disease or deficiency model to a more optimistic and salutogenic focus on strengths, assets and adaptive functioning, which gives new impetus to the development of preventive and therapeutic interventions [8].

Resilience is generally defined as a process by which individuals exhibit adaptive functioning despite encountering significant difficulties [9–14]. The term is used with reference to a positive outcome despite experiences of difficulties, continued positive or effective functioning in adverse circumstances, or recovery after a significant trauma [15]. It was in the 1980s that the dynamic nature of these development processes was identified [14–18].

The concept of resilience has been linked to positive development and good living patterns in longitudinal studies [8, 16, 19–21]. The characteristics of the individual are significant, as are the family environment and the larger social contexts [21, 22]. Risk factors may be attitudes, beliefs, behaviors or environmental conditions that adversely affect development. Poverty is, for example, a negative factor that is important for children’s health and development [7, 21, 23]. A review of longitudinal and cross-sectional studies has identified ten protective factors that play a role in resilience during childhood [24]: (1) effective parenting, (2) affiliation with other competent adults, (3) openness to other persons, especially adults, 4) good intellectual capacity, 5) a talent or skill worthy of the child and others, 6) self-reliance, self-esteem and hopefulness, 7) religious belief, 8) good socio-economic conditions, 9) a good school and other resources in the local community, and (10) good luck or success.

To capture these developmental processes, more system-based, ecological and transactional models have been developed (see, e.g., [11, 25–30]). A line of research examining the neurobiological aspects of resilience and PASTOR (the Positive Appraisal Style Theory of Resilience) is presented in a review article with the comments of 34 different research groups [31]. The model is based on the individual’s assessment of a situation as a central mechanism that leads to an emotional response. Another line of research seeks to identify the protective mechanisms that best explain positive development despite vulnerability [22]. Yet another line puts resilience into a social and cultural context [14, 32]. Here, resilience has been studied in eleven countries, with the development of methods of measurement [33].

In this project, the development of the RESCUR curriculum was based on available scientific evidence, using research on children’s resilience [1, 2]. Moreover, a theoretically well-developed instrument, validated and used in several countries, is used to measure how the level of resilience is affected by implementation of the curriculum.

### Children’s vulnerability

Children who are members a family with relatives with harmful use of alcohol often have a vulnerable everyday life. They run the risks of later ill-health and harmful use of alcohol of their own, and they constitute an exposed and vulnerable group in society [34, 35]. They have a multiplied risk of failure at school, suffer from mental health, and may develop their own harmful use of alcohol and drugs [35]. Therefore, the Swedish government has set as a priority area within the ANDT (alcohol, narcotics, doping and tobacco) strategy to protect children from their harmful effects [36]. The need for effective, well-founded efforts for this group is enormous. Ill-health is twice as high and abuse is 4–5 times as high among the children of addicts as in the rest of the population. There are huge economic savings to make, of 35 billion a year, if ill-health and harmful activity in the group can be reduced to the same levels as those of the rest of the population [37].

Mapping has shown the need for an enhanced child and parent perspective on dependency and addiction care. The children are also a strong driving force for parents with addiction problems seeking treatment and wanting to change their life situation [38]. At the same time, a study of 740,618 children in Sweden has shown that such a family situation is important for children’s schooling and future life [39]. The alcohol-related disorders of both mothers and fathers are associated with the lower school performance of their children at ages 15–16 years. Most of the statistical effects are attributed to the psychosocial circumstances of the family, such as parental psychiatric disorders, drug use and criminality, and the receipt of social or child-welfare interventions.

But we also know that far from all children can get access to the support available in society. Estimates have shown that between 4 and 5% of all Swedish children under the age of 18 live with a parent who has harmful use of alcohol/drugs [40, 41]. Studies at the Center for Health Equity Studies (CHESS) followed children born 1987–89 (*N* = 332,000) up to adulthood, and parents who were abusing or had addiction problems were identified via different registries. A total of 4% of all children had at least one parent who had been treated for harmful use of alcohol (2.5%) and drugs (1.5%) at childbirth [41]. In-depth analysis of the survey from 2012 showed that 56% of these parents want to talk about their parenting, but only 24% of the children living in families where a person was being treated for a dependency disorder was given formal support [42]. A minority of children receiving support already had problems of their own.

Another source of information on support for children in families where at least one parent has harmful use of alcohol/drugs is the report published annually by Junis (IOGT-NTO’s Junior Federation), now in its 12th consecutive year. It is based on surveys of Swedish municipalities, of which 73% participated in 2015 [43]. In 2005, 57% of municipalities had special support groups for children with a parent with harmful use. The proportion was 66% in the report published in 2015. However, in this report, the answers refer not only to support groups but also to individual support. And the number of participants in the various municipalities varied greatly. Conclusions from the different surveys are that there were major regional differences, and that there is a great need for increased efforts to strengthen the child and parent perspective in various parts of social services. Moreover, there is a great need for support for these children as only a small proportion receive formal support.

Accordingly, more actors are needed to offer support, not least non-government organizations. Value-based organizations have the advantage that they have more detailed knowledge of the problems. Junis is an NGO that works with children in Sweden. In addition to working with alcohol and other drugs issues, Junis is concerned with solidarity and democracy issues. Over the country, there are more than 12,000 members aged 6–15 years divided into about 175 local chapters. The organization is run by approximately 1300 voluntary trained leaders. In addition to Junis’s continuous weekly activities, every year throughout the country, a large number of camps are organized, as too are extensive activities in connection with the Christmas and New Year celebrations. Advocacy and education work supporting the needs of children who grow up in a family with harmful use of alcohol and drugs have been important part of Junis’s work plan for more than a decade.

In order to meet these challenges, the present research has been developed using three different complementary approaches: a universal approach through implementation of the curriculum in school classes, a selective approach through groups in the local chapters of Junis, and an indicative approach though groups recruited through the social services as support for the children of alcoholics.

## Methods/design

### Aims

The primary aims are to strengthen protective factors and promote childhood life changes for 7–14 year-olds who are relatives of people with addiction problems, and to contribute to the support that Swedish society gives to these children. We want to break the pattern of alcohol and drug problems being transmitted between generations. This entails a preventive intervention targeted at those who have an increased risk of developing addiction problems and other problems that are consequences of their situation. A secondary aim at the universal level is to promote protective factors among children of the same ages, and to prevent the development of drug addiction among children.

In order to increase our chances of achieving these goals, we are implementing the project as a collaboration between practitioners and researchers. Our goals also include increasing the relevance of the project, developing knowledge, and improving practical implementation. This can also provide an increased opportunity to disseminate experiences to other groups, and for other activities, after the method has been tested and evaluated based in the Swedish context and in three priority areas – social services, a NGO, and schools.

The research covers five research questions:

1. How can participants in the implementation of the program be recruited?
2. How can leaders be supported to enable the implementation of a high-quality program?
3. What is the significance of the program for children’s development and mental health?
4. Does the program work differently for different groups in relation to gender, social background, place of residence and age?
5. Does the intervention work differently at a universal, selective or indicative level?

The research questions will be studied through an implementation evaluation, using self-assessment forms, interviews and observations using an established “checklist”, as well as through an impact assessment, which has a randomized, controlled pre-post design, in which self-reports, interviews and adult reports of testing instruments are used. Our main target group consists of children aged 7 to 12 years who are in one of the organization’s schools, social services or the value-based organization IOGT-NTO Junior Federation, Junis. A secondary target group consists of the deliverers.

### Intervention method

The method of the present project, developed by experts in Europe, concerns giving children their own self-empowerment, strengthening protective factors, and, in the long run, their resilience. In Sweden, the material has been named “I want, I can, I dare”.

The methodology that will be further developed and evaluated in this project is called RESCUR (the resilience curriculum) aimed at increasing children’s resilience, i.e., children’s ability to cope with crises, changes and stressors without falling apart. The method is rooted in research on human resilience [1, 2].

It has been found that, when implementing foreign methods in a new country or culture, they rarely work [44], which provides the backdrop to this European cooperation. Instead of incorporating an American method, which in many cases has failed in Sweden [45–48], the starting point was to develop a method suited for European conditions (see https://www.rescur.eu). The present project mainly uses the leader’s/teacher’s guide developed for the target age group, i.e., children aged 7 to 12 years. In addition, there is a handbook for parents so that they can strengthen the work of promoting child protection factors at home and interact with the school in advancing child development. There is also a special common introduction in the material describing the theoretical foundation of the method. When these guides were written, all partners were aware that a particular focus would be on children in risk situations [2]. The guides describe very clearly how work with the children is going to be conducted, and there is a resource package with, for example, music, pictures, mindfulness exercises (recorded) to facilitate the implementation of the method. Instructions for teachers of children in three different age groups (4–5, 6–8 and 9–12 years) and guides for teachers and parents have been developed in seven different languages ​​(English, Greek, Italian, Croatian, Maltese, Portuguese and Swedish) [https://www.rescur.eu].

The curriculum is based on the following six themes, where each theme is divided into sub-themes. Clear goals are described for all exercises, which are the basis of the practical implementation of the method.

1. Developing communication skills: effective communication, and assertiveness.
2. Establishing and maintaining healthy relationships: healthy relationships, and cooperative skills, empathy and moral reasoning.
3. Developing a growth mindset: positive and optimistic thinking, and positive emotions.
4. Developing self-determination: problem solving, and empowerment and autonomy
5. Building on strengths: positive self-concept and self-esteem, and using strengths in academic and social engagement
6. Turning challenges into opportunities: dealing with adversity and setbacks, dealing with rejection, dealing with loss, dealing with family conflict, dealing with bullying, and dealing with change and transition.

The exercises are divided into three optional levels of difficulty (basic, intermediate and advanced level) to ensure that the exercises suit the target group and its needs.

Each RESCUR class begins with a brief mindfulness exercise. RESCUR has been piloted and evaluated in six countries with good results, and then further developed on the basis of lessons learned. The teachers who worked on the method with their students have been very satisfied, especially with the mindfulness part (mindfulness has been shown in a meta-analysis to be an effective, preventive method for children in the school environment) [49].

Due to the extensive material developed in RESCUR, we have chosen to constrain the part being evaluated. This evaluation will focus on themes 1, 5 and 6. The exercises are also divided into three optional levels of difficulty (basic, intermediate and advanced level) to ensure that the exercises fit the particular group you work with. Since RESCUR is a new method, being implemented at the same time as the evaluation takes place, and for comparable results, the intervention groups under investigation will only work at basic level.

The method is theoretically promising and builds on principles of successful preventive and promotional work, but it has never been tested or researched in Sweden. We believe it can be an important element in the support offered to children, and their relatives and teacher/group leaders. However, it should be stressed that it is not a way of replacing actions at the structural level carried out by municipalities or county councils; successful promotive and preventive work needs to be implemented on many levels and by many different actors. RESCUR is a method to complement other efforts made by society in to provide children and relatives with improved protective tools so that they, in case of difficult situations, can not only handle the situation but also be strengthened. In other words, it is about helping children to get as good life chances as they possibly can.

The RESCUR program has a training component for the teachers and group leaders who will implement the curriculum. The training lasts 3 days. First, there is a two-day introduction to the theory and methodology of RESCUR, which also includes practical exercises. The teachers and group leaders are supervised through a monitoring visit to the different sites every sixth month for RESCUR in Sweden. The supervisors also observe the implementation and document results on an implementation checklist. There is also a one-day educational session for follow-up.

### Research design and methodology

The project has been divided into three phases. The first phase involved planning and conducting a preliminary study, including the application to the research ethics committee (first year). We started the project, formed a project team and appointed a project manager, worked on the manuals to make them more user-friendly, gave information about and marketed the project among organizations and policy-makers, recruited participants for the evaluation, finalized the project and research plan, including all measuring instruments. The Ethical Review Board approved the research project (EPN dnr 2016/460).

The project is based on close collaboration between Junis and the research group, with a clear division of work and responsibility. The purpose of Junis’s activities is to provide health promotion for children aged 7–15 years in an encouraging environment where the child’s self-esteem and self-confidence grow together with secure and safe adults. The organization will be responsible for planning and implementing the program in all groups. The research group is responsible for the scientific study of implementation and effects of the program for the participants in groups and school classes who are part of the intervention and control groups. The research consists in a randomized, controlled, longitudinal survey of the RESCUR program. This means taking baseline measurements before the intervention begins, and follow-ups at 6 months, 1 year, and then 2 years.

The second phase involves implementing the program “I want, I can, I dare” (RESCUR) in Junis’s group activities, in groups throughout social services and in school classes in different municipalities and across the country (i.e., in different residential areas). The third phase involves analysis and reporting on the project’s implementation and its effects.

### Design

The project will be carefully evaluated from two perspectives: implementation and impact. In order for a theoretically promising method to work at all, the method must have been implemented effectively and correctly. Inadequate implementation leads to poor or no results for what would otherwise have been an effective method. We will study the implementation through both self-assessment forms, reported by group leaders when the program has been used for half a year, and observations made according to a formalized checklist. Implementation of the method is fundamental to properly evaluating the effects of the method, which is the second part of the evaluation of the project. At the same time, we are also interested in directly studying the extent to which it is possible to implement the method as intended. This is one of the research issues in the project.

The effects of the method on protective factors and resilience will be measured using a randomized, controlled pre-post follow-up design with two types of groups. We will study the effects both statistically and with regard to practical relevance [62]. We will use our waiting list to create a control condition. In this way, we can use the principle of giving everyone who so wishes have the chance of receiving the intervention in combination with increasing its internal validity. The waiting list group is initiated 1 year after baseline measurements have been taken. We take a pre-measurement before the intervention begins among the participants (even adult reports will be collected), and then take measurements after one semester, 1 year, and 2 years (Table 1). Adult reports will be collected at baseline and at the measurement after 1 year. On other occasions, only the children’s self-reports will be collected. By collecting data from two different informants for each child, we ensure that we get information on each child to facilitate analysis of data reliability. In addition, we will probably get a “truer” picture of the child’s behavior.

**Table 1:**
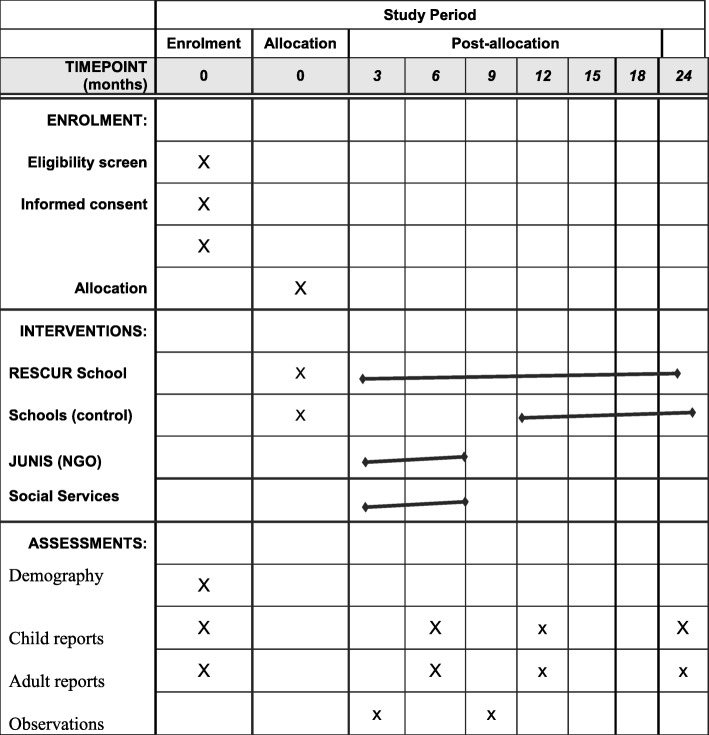
Schedule of enrolment, interventions, and assessments

The controlled study has three different arms. In the case of schools, we cluster-randomized participants to one of two conditions: (A) intervention schools or (B) waiting list or control schools. Randomization has been done at school level. Thereafter, the baseline measurements were performed in both conditions, and, after that, the intervention group began its activities, and measurements were taken as described above (Table 1). One year after the baseline measurement intervention group (A), the waiting list group began its program. Measurements are finally to be made a year later.

In the case of Junis’s groups, it was not feasible to do a cluster randomization at the managerial level as the involvement of local chapters was more difficult to achieve. The groups will be led by regular Junis volunteers.

As for the social services group, we will have the same arrangement, but no waiting-list control condition. Here, a new round of groups is planned for the second year. On the other hand, we will have a follow-up measurement of the first wave’s intervention groups in year 3 for both the Junis and the social services groups. The groups will be run by social workers.

However, it will be possible to compare developments in these two study arms with the data collected in the control schools.

### Participants and recruitment

A calculation of statistical power has been made for the interventions. For the universal intervention, there is an effect size of d = 0.3 and a power of 0.8, and the corresponding statistics are expected to test a group size of 176 in each group. The recruitment of schools for the evaluation of RESCUR has been completed. These schools have been recruited from different parts of Sweden, and in both urban and rural areas. There are 8 intervention schools in five municipalities, including 10 classes in school year 1, and 8 classes in school year 4. The intervention group includes 394 children. There are 9 school in the waiting list control schools in six municipalities, including 10 classes in school year 1, and 9 classes in school year 4. The waiting list controls include 401 children. The number of classes in which RESCUR is carried out in the municipalities is larger than necessary to carry out the project based on an evaluation or “power perspective”. Oversampling, however, is a common and good strategy to deal with any delays, etc. from the project, and thus to reduce threats to the implementation of the project. Participants are randomized to the intervention and waiting list/ control groups based on residence and school. The planned survey is regarded as having adequate statistical strength.

The project group has trained 23 teachers in the intervention schools. In March 2018, 20 teachers in the waiting list control schools will be trained in the RESCUR intervention.

A power analysis for the interventions in social services and the NGO has been performed. For the selective groups, an effect size of d = 0.5 and power of 0.8 is calculated, which results in a double-sided statistical testing of group differences (alpha = 0.05), and a group size of 64 children in each of the two groups. The planned size allows for a certain amount of dropout in the study.

Recruitment for the evaluation of RESCUR in the social services groups is ongoing. At present, several social-services units have expressed an interest in being included in the evaluation. We will recruit participants to 8 groups with approximately eight participants for the first year. Here, there will be no randomization, but a second round of groups is scheduled for the second year. It provides a total of 128 children over the 2 years. The planned size of the sample in social services is less, since previous research has clearly shown that interventions in targeted or selected groups have a significantly higher effect size than those in universal groups (i.e., the school). The age of participants in the social services groups will be 7–9 and 9–12 years. During 2017, RESCUR groups have started in social services in five municipalities, including 42 children. Fifteen group leaders from six municipalities have received the RESCUR training so far.

For groups within Junis, 22 leaders from ten different districts have been asked to participate. We plan to have a total of 16 groups of approximately 8 participants in the Junis group. It provides 128 participants. The age of the participants will be between 7 and 12 years. Eight Junis leaders from 5 municipalities have been trained, but so far only one group has started to use the RESCUR intervention.

An important question to answer in the project is how the recruitment of participating actors as well as children is facilitated in the three types of organization. Another important question is whether the RESCUR method works equally well for children in the three types of organizations.

### Data collection and outcome measurements

#### Implementation study

Data will be collected on implementation through self-reports from group leaders/teachers on the implementation of the method and how they experienced it. These self-reports will be completed together with the leaders/teachers implementing the method after 6 months.

Measures of the extent to which the children have been exposed to the method and how well the leaders implemented the method will be taken. Barriers and success factors in implementation will also be assessed by interviews with a subgroup of deliverers (*n* = 10). Observations will be performed using implementation checklists twice a year as the method is conducted to study the implementation of the method. The aspects of the implementation of the program that will be the focus of the evaluation are: dose or exposure, program fidelity, customization, quality of the implementation of the program (quality of delivery), and how participants react to the program and its implementation. Implementation is a key aspect of prevention and health promotion because it affects the results achieved by the intervention [50, 51].

#### Effect study

To measure the effects of the program, we will use child reports (self-reports) and adult reports. Data from the children will be collected to enable analysis of program effects and effects on children’s resilience, health and well-being. These factors have a strong connection with the harmful use of alcohol/drugs, and the outcome measurements can also be seen as intermediate effects in relation to longer-term outcomes (which must be measured when participants are several years older) and that concern the use of ANDT. We will investigate how the method works for different groups based on age, gender, place of residence, and problem levels. During the planning phase, a balance has been made between obtaining data that is age-appropriate for the children on the one hand, and the ability of researchers to perform more sophisticated analyses on the other.

#### Self-reports from the children

All measures have been translated into Swedish in previous research projects, and the psychometric tests have been shown to work well in the age groups we are evaluating. The instruments that we will use are the following:

- Child and Youth Resilience Measure (CYRM) [52]. In order to make the evaluation as change-sensitive as possible, we will use the variant with a 5-point Likert scale, instead of the 3-point.
- How I feel (HIF) [53] is used for older children, and further development of the instrument for younger ages is underway in our EU Comenius Project European Assessment Protocol for Children’s SEL skills [How one feels (HOF)].
- Health, psychiatric symptoms, somatic symptoms and health behaviors from HBSC (Health Behaviour of School-aged Children) [54, 55],
- Mastery [56]
- Demography (gender, age, housing situation, family structure).

#### Reports from adults

At least one adult per child will fill in instruments about the child’s behavior. For the school group, this will be the child’s teacher. For the social services group, it is primarily a parent, or secondarily a social worker, who will respond. For the Junis groups, leaders are assessing the behavior of the child. The adults will fill in two instruments. Both instruments have previously been translated into Swedish, and both have been shown to have good psychometric properties.

- Child and Youth Resilience Measure - Person Most Knowledgeable (CYRM) [33, 57]. In order to make the evaluation as change-sensitive as possible, we will use the variant with a 5-point Likert scale, instead of the 3-point. This is exactly the same measure that the children themselves may fill in.
- Strengths and difficulties questionnaire (SDQ) [58, 59]. SDQ is an international and widely used instrument, and is also a widely used measure in Sweden.

The school classes and groups who will fill in surveys will receive as small economic token in appropriate forms from the research project.

### Data management and analysis

All collected material from surveys, observations and interviews will be encoded before analysis. The code key, with the researcher’s name and code number, will be stored separately in fireproof locked cabinets, which only research director Therése Skoog has access to. All collected material will be stored for at least 10 years in other fireproof archives, as required by law. All primary data will be stored at Göteborg University. Because the project has a longitudinal design, the research participants are not anonymous. However, it is of the utmost importance that the principle of confidentiality is followed. The names, addresses or the like of the children, parents or co-workers will not be read-in without being treated confidentially and only for this project. The results are only reported at group level, which means that individuals cannot be identified.

The research group has monthly meetings together with the implementing organization in order to facilitate the smooth running of the intervention study. In order to answer the five research questions, both quantitative and qualitative analyses will be applied in this intervention study, which uses a mixed methods research approach. The quantitative data will be managed using SPSS as a tool for analysis. In the randomized trial, we will use latent change models to examine the changes in child outcomes over time. To obtain unbiased estimates despite attrition, we will employ intention-to-treat analysis. Cohen’s *d* effect sizes were computed to estimate the effects of the programs compared with the wait-list condition while taking the initial level of each outcome measure into account. The social services and the NGO groups will be compared with controls matched from the waiting list controls. Mediating and moderating factors will be analyzed with regard to the primary outcomes.

The interviews will be transcribed and analyzed by qualitative content analysis. All sources of data will be used in the implementation study, where the inferences will be drawn using mixed-methods procedures.

## Discussion

The protocol of this research project has shown how the planning and development of the research make it feasible to answer the five research questions.

1. How can participants in the implementation of the intervention be recruited? The question is answered by analyzing the interviews with the leaders and researchers developing the methodology and the framework of the study. Analysis of the written documentation that the research group continuously makes during the recruitment work is also important.
2. How can leaders be supported to enable implementation of high quality intervention? This question will be approached through the documentation of the tutorial process as well as the analysis of interviews with teachers and group leaders. The support and supervision of the people implementing the method is of great importance for the results [60, 61].The project can contribute to filling knowledge gaps by implementing the planned intervention in school and in recreational activities, where there are no studies available according to the Swedish Agency for Health Technology Assessment and Assessment of Social Services’ (SBU) review of controlled prevention of child and adolescent abuse [62]. By planning for the long-term follow-up of initiatives, future preventive effects can also be made.
3. What is the importance of the intervention for the development of children and their mental health? Mainly statistical analyses of data from pre- and post-measurements. Analysis of interviews with children and interviews by leaders will complement the statistical analysis.
4. Does the intervention work differently for different groups in relation to gender, social background, place of residence, and age? Comparisons are made between the results of pre- and post-measurements, with child and adult reports for the different groups.
5. Does the intervention work differently at universal, selective and indicated levels? The answer will be based on before and after measurements, with child and adult reports and statistical analyses. Comparisons of effect sizes will be made between the arms of the study (voluntary organization, social services, and school). Analysis of leader interviews and observations will complement these comparisons.

Good measuring instruments are needed to further develop the field of study. In an overview, it was found that there was no gold standard among the 19 scales used for measuring resilience [63]. According to one research group [64], a combined assessment is needed, including assessment of vulnerability (severity, chronic or not, ecological level, child’s perception of causes, as well as cultural and contextual relevance), of resilience (with an individual dimension – personality and thinking, and with a contextual dimension), and of contemporary and cultural aspects. The contextual dimension covers all levels of vulnerability, such as the availability of individual, family, social and political resources. It covers strategic use of biological, emotional, psychological, spiritual, social and political resources. Moreover, the contextual dimension also includes dynamic elements such as positive feedback on individual coping strategies from family and society, and adaptability in the environment to the needs of individuals, families and society [64]. The chosen measure of resilience, the Child and Youth Resilience Measure, is a promising newer tool that has been developed to cope with the challenges to measure resilience in different settings in an age-appropriate way.

As this research deals with children, ethical considerations are of the utmost importance. It is important for this research project to protect both the generation of knowledge [65] and the individual [66]. This means taking consent, confidentiality and utility requirements into account. There are several ethical challenges in the project that have to do with the groups’ vulnerabilities. Researching children means that ethical issues must be clarified. This applies, for example, to access, power and consent [67]. It is also important not only to ask children about their views, but also to make sure their views really count [68]. Research should be done with children instead of just on or about children [69]. Children’s views may differ from what adults think children think [70].

In order to give consent, it is important that children receive very detailed and age-sensitive information, especially about the right to say no to participation [70, 71]. Children and adolescents must be protected in research, which means that the risks of ill-health or injury must be minimized. This means that we need a strategy for how the child or youth will be supported if problems arise [71]. If an individual in the project is under the age of 15, informed consent from a parent is required for participation in the research [72]. In order to comply with research ethics, we will seek active consent from the children before the evaluation starts. After the children have received information about the project and the evaluation, including ethical principles, they can accept or refuse to participate in the evaluation. The child’s response to participation in the research will not affect their participation in the program and how they are treated or responded to otherwise.

The study includes children who are in an exposed situation. Thus, it is extra important to conduct the project so that these children will not be harmed. We will follow all ethical requirements for the implementation of the project. This applies both to the implementation of the method and the evaluation. Well-trained and experienced staff will deliver the method. The work will be carefully monitored through self-assessment forms and observations by the project management team. Conventional working methods for conducting research with children in Sweden will be followed [73]. We take these measures to reduce the risk of children being harmed in any way.

At the same time, it is of the utmost importance that particularly this group of children, who run a high risk of developing their own problems, get interventions that are tested and effective. It is also important that the child’s own perspective is considered. At the same time, it is important to distinguish between children’s perspectives and the perspectives of adults on the child [74].

Our main target group consists of children aged seven to twelve. Because the children are so young we will also obtain active consent from the children’s parents. The main principle is to collect parental consent in writing. In cases where we fail to communicate with the parents by letter, they will be contacted by telephone and asked for their active consent for their children to be included in the study. Thus, we use dual active consent, from both the child and the parent. Both parties must accept study participation for the child to be included in the study. Our secondary target group includes program deliverers, who give active consent.

Implementing this type of research with the highest ethical standards is a key concern, as well as implementing the RESCUR material in an efficient way together with participating children, parents, teachers and group leader. We consider the potential of the project to benefit children in general, and children in vulnerable positions in particular, to be high.
